# Large gap Quantum Spin Hall Insulators of Hexagonal III-Bi monolayer

**DOI:** 10.1038/srep34861

**Published:** 2016-10-07

**Authors:** Qunqun Liu, Ying Dai, Yandong Ma, Xinru Li, Tiejun Li, Chengwang Niu, Baibiao Huang

**Affiliations:** 1 School of Physics, State Key Laboratory of Crystal Materials, Shandong University, Jinan, 250100, People’s Republic of China; 2 School of Computer Science and Technology, Shandong University, Jinan, 250100, People’s Republic of China

## Abstract

In the present work, we demonstrate that both GaBi_3_ and InBi_3_ monolayers are Quantum Spin Hall insulators. Here, the electronic band structures and edge states of the two novel monolayers are systematically investigated by first principle calculation. Our analysis of the band inversion and Z_2_ number demonstrate that both GaBi_3_ and InBi_3_ are promising 2D TIs with large gaps of 283meV and 247meV, respectively. Taking GaBi_3_ as example, it is illustrated that the edge states are impacted by SOC and finite size effect. In addition, it is found that the compression and tension totally affect differently on the edge states. Finally, the electron velocity is studied in detail, which is highly important in the manufacturing of spintronics device.

Recently, topological insulators (TIs) have been paid much attention. Different from the ordinary materials, which can be categorized either to insulator or conductor, TIs are of conducting surface states which are protected by time reversal symmetry^1^,^2^,^3^,^4^. For 3D TIs, it has been found that the scattering of electrons on two dimensional surface can happen at any angle less than 180°^5^,^6^. One method to get rid of this shortcoming is to reduce the surface of 3D TIs and thus 2D TIs are great choices.

Thanks to the appearance of graphene^7^, the study of 2D materials has grasped much attention both in experimental and theoretical fields^8^. Unfortunately, graphene is limited when put into use due to the unobservable narrow bulk gap (~10^−3^meV)^1^,^9^, so it is necessary to look for 2D TIs with much higher SOC strength. Among the heavy elements, Bi is a familiar constituent of many TIs. Recently Bi(111) monolayer has been investigated to be 2D TIs with much larger bulk gaps (560meV)^10^. Besides, Bi(111) has been successfully synthesized on Si(111) substrate^11^,^12^,^13^, and this makes the practical use of Bi(111) highly possible. Recent simulation research found that Bi(111) monolayer doped by III element (Ga, In, Tl) open the energy gap to a much higher level^14^,^15^. All these researches indicate that III element and Bi form a most suitable match. Unfortunately, most of these researches are limited to XBi (X = Ga, In, Tl) form, which totally lack the other possible X and Bi compounds, so it is necessary to look for other X-Bi distribution in order to give a complement research of the III-Bi topological nontrivial materials.

In the present work, we propose a nontrivial topological structure XBi_3_. This structure is obtained by modifying the 2 × 2 primitive Bi(111) cell using X atom(X = Ga, In, Tl). The XBi_3_ distributions which do not have the hexagonal symmetry are also considered, but the results indicate they are not stable. Also, the total energy of XBi_3_, XBi and Bi film show that E(XBi_3_) < E(XBi) + 2E(Bi), which means that the formation of XBi_3_ film is thermodynamically stable. Besides, the heterostructure of XBi_3_/BN is fully optimized. The result shows that XBi_3_ preserve its structure in a large degree, which means that XBi_3_ can be realized on BN substrate. Based on first principles, we find that both GaBi_3_ and InBi_3_ are promising candidates for 2D TIs, with large energy gaps being 283 meV and 247 meV respectively. It can be clearly seen that the band inversion appears both in GaBi_3_ and InBi_3_. The Z_2_ number and gapless edge states further conform their topological characters. To understand the formation of the gapless edge states, a series of nanoribbons with different width are constructed. It is found that the competition or cooperation between SOC and finite size effect are of great importance to the formation of the gapless edge states. Finally, the energy bands of GaBi_3_ under external strain are examined in detail. The results show that the topological property is robust under external strain from −8% to +8%, but their corresponding nanoribbon edge states change differently under compression and tension. The velocity of electron changed dramatically due to the change of the external tension. All these researches indicate that both GaBi_3_ and InBi_3_ are promising candidates to manufacturing non-backscattering electronic devices.

## Results and Discussion

**Figure 1:**
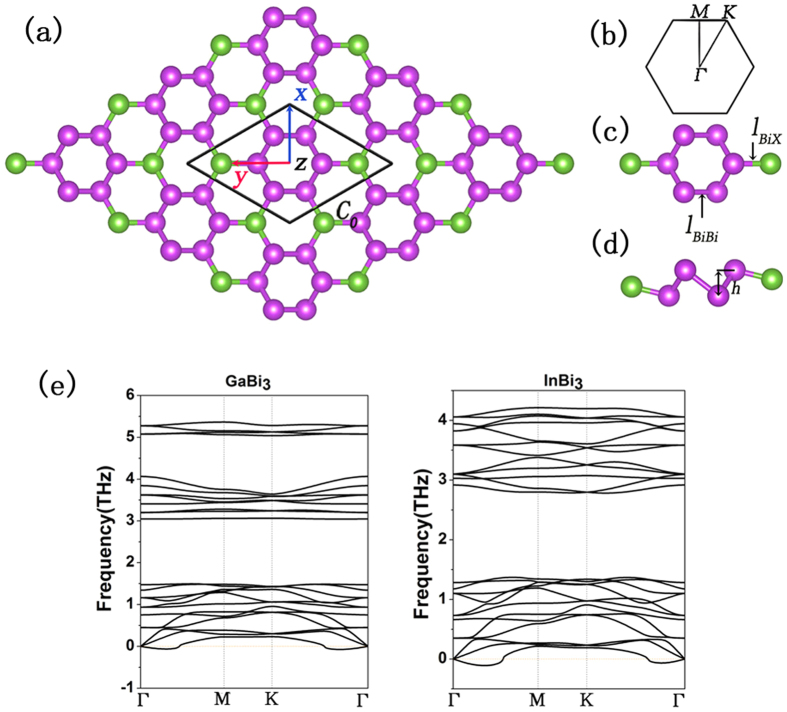
(**a**) Geometric structure of XBi_3_ monolayer. The purple balls represent Bi atoms and the green balls represent X (X = Ga, In, Tl) atoms. (**b**) First Brillioun zone of XBi_3_. (**c**,**d**) Top and side view of geometric structure of XBi_3_ primitive cell. (**e**) Phonon spectrum of GaBi_3_ and InBi_3_ (TlBi_3_ is omitted for its metallic character).

The XBi_3_ has hexagonal lattice with eight atoms per unit cell, as is shown in Fig. 1a,c. The optimized structure parameters of all XBi_3_ are listed in Table 1. The lattice constants of XBi_3_ are 8.824, 9.121, 9.170 Å, respectively. It is clearly shown in Table 1 that the lattice constants, bulking heights and bond length l_BiX_ increase in accordance with the electronegativity: c_0_(Ga) < c_0_(In) < c_0_(Tl); h(Ga) < h(In) < h(Tl); l_BiGa_ < l_BiIn_ < l_BiTl_ while l_BiBi_ remains almost the same. Figure 1 displays that the primitive cell in the structure consists of two hexagons, with the Bi-hexagon nested within the X-hexagon. Although the phonon spectrums show imaginary mode between Γ and M points which corresponds to 4 nm, the fully optimized nanoribbons (9.8 nm for GaBi_3_ nanoribbon and 12.0 nm for InBi_3_) still keep their original shapes on a large scale, despite a small deformation on the sides which can be totally neglected. Moreover, the band spectrums of nanoribbons preserve clear Dirac cones, which means that their flakes can exist as long as their width are limited appropriately^16^,^17^. After all, the structure can never be made infinity large in practice, with 9.8 nm and 12.0 nm sufficiently large for experimentation. All these experiments make the synthesis of the XBi_3_ structure highly possible and we believe that these analysis provide sufficient arguments for the stability of the XBi_3_ structure.

**Figure 2:**
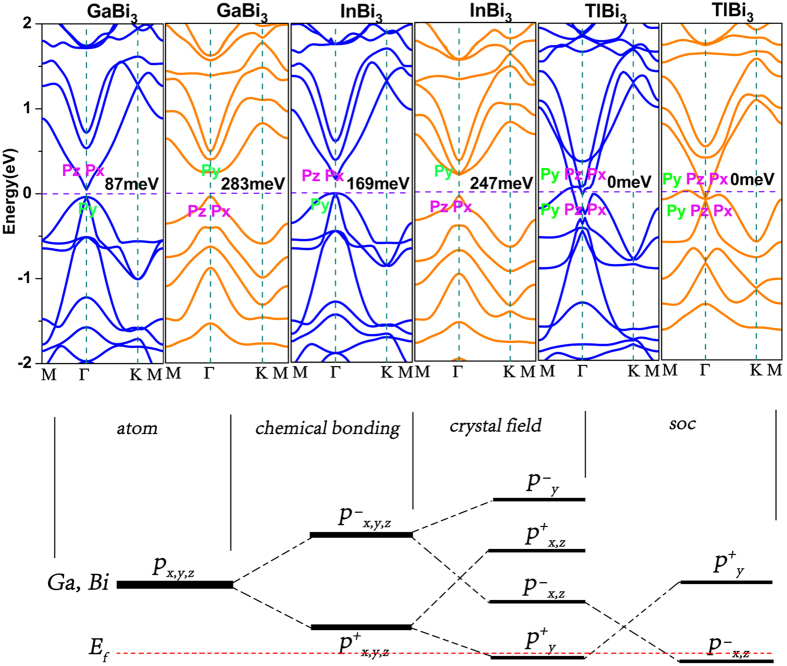
Upper part: The electronic bulk band structures of GaBi_3_, InBi_3_, TlBi_3_. The blue lines and red lines represent the band structures without and with SOC, respectively. The horizontal purple dashed line indicates the Fermi level. Lower part: The evolution from atomic p-orbit of Ga and Bi to VBM and CBM at Γ point for XBi_3_, fermi level (E_f_) is indicated by the red dashed line. The parities of the states at Γ point are denoted by + and −.

The upper part of Fig. 2 shows the energy bands for XBi_3_ with and without SOC. It is illustrated that the energy bands for GaBi_3_, InBi_3_ are similar to each other without spin-orbit coupling (SOC). They all have two degenerating energy bands below the Fermi level and are all semiconductors. As for TlBi_3_, the Fermi level pass through energy bands, making TlBi_3_ semimetal. When the SOC is taken into consideration, great changes take place. For GaBi_3_ and InBi_3_, the energy degenerating below Fermi level is lifted. As for TlBi_3,_ it remains a semimetal character, although the energy bands became more regular. The analysis above demonstrate that SOC have great influence on the energy spectrums for all the XBi_3_ monolayer, and there might be band inversion occurring, which could induce a topological phase transition.

Band inversion is an important precursor for materials to be TIs^4^,^18^. After analyzing the components of the orbitals at Γ point near the Fermi level with and without SOC, it is shown in Fig. 2 that with regard to GaBi_3_ and InBi_3_, the energy bands above Fermi level at the Γ point mainly come from P_z_ and P_x_ orbitals and the two degenerated bands below the Fermi level mostly originate from P_y_ orbitals. As for TlBi_3_, the energy bands nearly the Fermi level mainly come from P_z_, P_x_ and P_y_ orbitals. When SOC is taken into consideration, for GaBi_3_ and InBi_3_ the energy bands above the Fermi level are mainly contributed by P_y_ orbitals, while the bands below the Fermi level mainly contain P_z_ and P_x_ orbitals, which are exactly inversed compared with that when SOC is not applied. For TlBi_3_, the energy bands remain gapless, and its bands near the Fermi level still mainly consist of P_z_, P_x_ and P_y_ orbits, which is the same as that without SOC. From the analysis above, it is firmly demonstrated that band inversion occurs in GaBi_3_ and InBi_3_ monolayer under the effect of SOC. For TlBi_3_, it is difficult to know only from the band inversion method whether there is topological phase transition due to its metallic character. To get a further understanding of the origin of the nontrivial topological order, the evolution of atomic p-orbits of Ga and Bi to the VBM and CBM at Γ point of GaBi_3_ and InBi_3_ is displayed in the lower part of Fig. 2. A brief analysis shows that all these states are derived from both Ga and Bi. The electron configurations of Ga and Bi make sure that the bonds are composed of p-orbits, with the s-orbits deeply locate below Fermi level. From Fig. 2 it is shown that due to the chemical bonding the p-orbits of Ga and Bi split into the bonding |p^+^_x,y,z_> and antibonding |p^−^_x,y,z_> states. The analysis shows that the p_x_ and p_z_ orbit are across the bonds while the p_y_ orbit is along the bonds, leading to considerable difference between p_y_ and p_x,z_ owing to crystal field, which split |p^±^_x,y,z_> split into |p^±^_x,z_> and |p^±^_y_> states, with the |p^+^_y_> and |p^−^_x,z_> below and above E_f_, respectively. Then the order of |p^+^_y_> and |p^−^_x,z_> respect to E_f_ is inverted at Γ point due to the consideration of SOC. The inversion of band order at the Γ point is a strong indication for the transition of nontrivial topological phase. For further identification, more arguments are shown below.

Since the structure conserve the inversion symmetry, the Parity method can be used to further convince the nontrivial topological phase in XBi_3_ monolayers^19^. Using the formula,


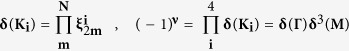


where ξ = ±1 is the parity eigenvalue and N is the number of the occupied bands. The Z_2_ topological invariant (ν) have two values: ν = 1 implying a nontrivial phase and ν = 0 implying a trivial phase. From the parity of occupied bands at Γ and M points it can easily obtain that all three structures show a nontrivial phase: ν(GaBi_3_) = 1; ν(InBi_3_) = 1; ν(TlBi_3_) = 1.The nontrivial phases for GaBi_3_ and InBi_3_ correspond to the band inversion analysis mentioned above.

**Figure 3:**
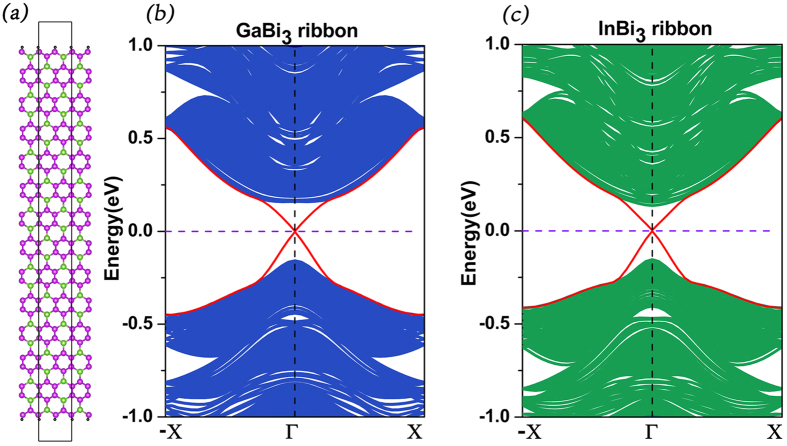
(**a**) The geometric structure of XBi_3_(X = Ga, In) nanoribbon. (**b**,**c**) Electronic band structures of GaBi_3_ and InBi_3_ nanoribbon. The blue or green shadow part represent the bulk energy spectrum, the red line represent the edge states. The widths are 9.8 nm and 12.0 nm for GaBi_3_ and InBi_3_ respectively.

Topological insulators are best known for their topological protected edge states^1^. Using slab model, we introduce zigzag edges on GaBi_3_ and InBi_3_ monolayers to show their marking edge states. The edge atoms (Bi) are passivated by hydrogen atoms to eliminate the dangling bonds. The zigzag nanoribbons are infinite along y direction, while the vacuum slabs of 15 Å are implemented along x and z directions to avoid interactions between adjacent nanoribbons. In Fig. 3, it is clearly that the gapless edge states (red lines) appear in the bulk gap and are linear at the Γ point, which conforms the topological nontrivial property of both GaBi_3_ and InBi_3_.

**Figure 4:**
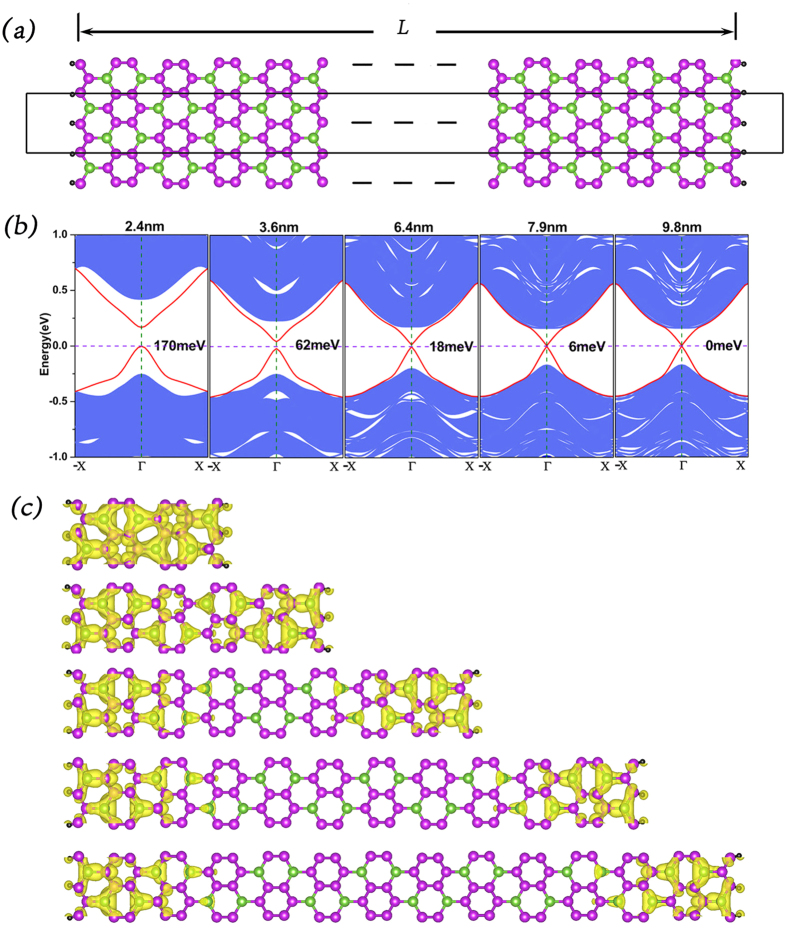
(**a**) Nanoribbon of GaBi_3_ **(b)** Electronic band structures for the GaBi_3_ zigzag nanoribbons of 2.4 nm, 3.6 nm. 6.4 nm, 7.9 nm and 9.8 nm, respectively (**c**) Real space charge density distribution of the edge states at Γ points.

The linear dispersive edge states are the most marking phenomena in TIs. However, their formation are largely affected by SOC strength and finite size effect^20^,^21^,^22^, for example, element doping and strain can change SOC strength while width variation contributes to the change of finite size effect^23^,^24^. In view of these arguments, we construct a series of nanoribbons of GaBi_3_ with zigzag edges. The widths of the nanoribbons are L = 2.1 nm, 3.6 nm, 6.4 nm, 7.9 nm and 9.8 nm, respectively. Their band spectrums and corresponding real-space charge density distribution of edge states at Γ point are shown in Fig. 4b,c. It is shown that when L = 2.1 nm, there is a large energy gap between the edge states of about 170 meV, which is much higher that room temperature (~26 meV). From its corresponding real-space charge density distribution displayed in Fig. 4c, it is easy to see that the charge density of the edge states distributes almost everywhere along the nanoribbon. As L increases to 3.6 nm, the energy gap decreases to 61.5 meV and there appears a vague dividing line between the charge density of the edge states. When L increases to 6.4 nm, the magnitude of the energy gap decreases to 17.5 meV and there appears a no-charge-density middle part. When L reaches to 7.9 nm, the edge gap decreases to about 5.5 meV which makes no matter compared with the room temperature, and when the width exceeds 9.8 nm, the edge states show a perfect Dirac cone.

Finite size effect are inevitable factors which influence edge states in practical utilization. The increasing of the finite size effect opens the edge states and vice versa, while the increasing of the SOC strength closes edge states and vice versa. It is widely known that SOC strength can be tuned through external strain, which is definitely unavoidable when the 2D materials are synthesized on a certain substrates, for example, the Si(111) substrate, and even threats the topological insulating character of the 2D materials^25^,^26^,^27^,^28^. For the reasons presented above, we give a detailed research about the influence of the external strain on the 2D TIs and their corresponding edge stats. After a detailed examination of the band inversion, it is shown that all these strained monolayers still preserve the topological insulating character, with energy difference of about 810 meV at Γ point when strain changes monotonously from −8% to 8%.

**Figure 5:**
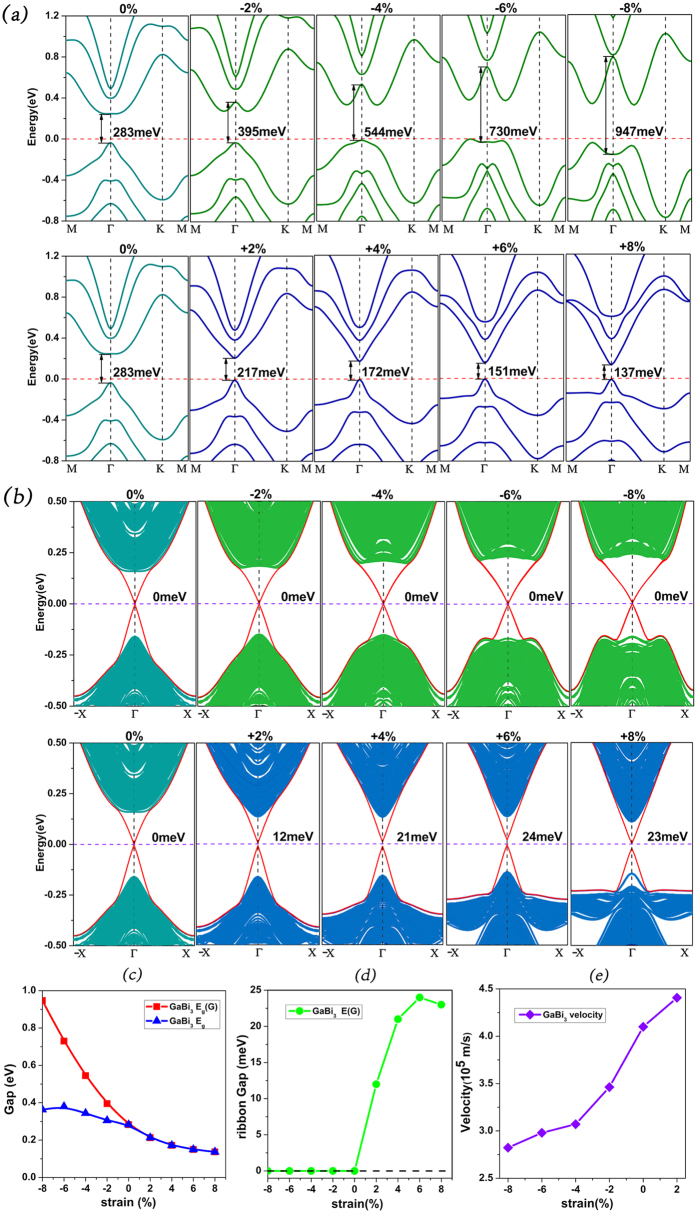
GaBi_3_ monolayers under strain from −8% to +8% for **(a)** bulk energy spectrums according to strain **(b)** edge states according to strain. **(c)** The variation of the bulk direct gap E_g_(Γ) at the Γ point and the indirect band gap E_g_ as a function of external strain. (**d**) Variation of the ribbon edge gap as a function of external strain. (**e**) Electron velocity near Fermi level at Γ point with the strain from −8% to 2%.

Although this structure has a robust topological insulating character against external strain, its edge states may still be influenced. So for each strained GaBi_3_ we constructs its corresponding nanoribbon of about 10.0 nm respectively. The width of the nanoribbons will change according to the applied strain. The positive in abscissa means tension while the negative means compression. In Fig. 5b, it is shown that the energy gaps between the edge states increase monotonously from 0 meV to 24 meV when the strain varies from 0% to +6%, then the gap decreases from 24 meV to 23 meV when the strain varies from +6% to +8%. This is because the decrease of the bulk gap with tension shown in Fig. 5a gives rise to the spreading of the edge-state wavefunctions into the bulk and thus enhance the overlap between edge states localized at opposite edges, with a consequent gap opening (and increase). When the strain reaches +8%, the gap anomalously reduces by 1 meV. The reason is that the change of finite size effect becomes strong enough to neutralize the contribution given by SOC and even dominate the variation of the edge gap. All these show that the competition between SOC and finite size effect is a crucial factor for the formation of the protected linear edge states, which is very important in practice owing to the inevitable external strain. As the strain changes from 0% to −8%, the velocity of bulk state electron increase while the velocity of edge state electron decrease. This is because the interaction between atoms enhances under external compression, resulting in the increase of the SOC strength. This situation leads to enhanced band-inversion and thus the band dispersion increases, which is marked by the increased Fermi velocity of the bulk state. However, as compression increase, the Fermi velocity of the surface state decrease. This phenomenon agrees with the situation of Sb doped Bi_2_Te_3_, which states that the increased SOC strength is the key ingredient which leads to the decrease of the Fermi velocity of the surface state and vice versa^23^.

Here appears another interesting phenomenon. When the ribbon is stretched to some point, as we have studied above, the influence of finite size effect will prevail, leading to a small reduction of 1 meV from +6% strain to +8%. So one may have a rational guess that when compression is applied, these two effects may have a chance to be equally matched at some point, i.e. a turning point, similar to the turning point at +6%. Apparently, no sign of gap opening occurs in Fig. 5d. The explanation of this mismatch lies in the changing pattern of E_g_(Γ). From Fig. 5a,c it is shown that although the range of strain is the same for both tension and compression, the changing for E_g_(Γ) differs greatly. For tension from 0% to +8%, E_g_(Γ) gives a reduction of only 146 meV, while for compression from 0%to −8%, E_g_(Γ) gives an increase of about 663 meV, which is almost five times the size compared with the reduction when tension is applied. The reason for the changing difference between tension and compression can be understood using the simple Lennard-Jones potential(L-J potential). Although the L-J potential is used to describe the two-body interaction, we can draw a rough treading that the interaction caused by compression changes much faster than that caused by tension, provided that both situations have the same variation of the distance between atoms. The energy gap at Γ point opens because of band anti-crossing caused by SOC^29^,^30^. With the applied external strain, the hybridization of atomic orbits are modified, which changes SOC to different extent^24^. So, if the finite size effect have a small opportunity to prevail the influence from SOC when tension is applied, it totally has not the slightly chance to compete with SOC when compression is applied.

## Conclusion

In summary, using first-principles, we illustrate that GaBi_3_ and InBi_3_ monolayers are 2D topological insulators with bulk energy gaps of about 283 meV and 247 meV, respectively. As an example, GaBi_3_ shows finite size effect greatly influence the edge states. After a detailed research for the bulk energy bands and edge states with the external strain applied, we illustrate that while this kind structure has a robust topological insulating character against external strain, the SOC strength is dramatically changed, and this leads to appreciable variation to the edges states. The result shows that although compression does not threat the topological character of the material, it surely decelerates the electrons of edge states, while we can get a higher speed of these electrons if the tension is applied appropriately. All these come to the conclusion that these hexagonal structures are promising candidates for high-speed spintronics device.

## Methods

Our first-principle calculations are performed using the plane wave basis Vienna ab inito simulation package known as VASP code^31^,^32^, the projector-augmented wave (PAW) method is used to describe the electron-ion potential. The electron exchange-correlation functional is treated using a generalized gradient approximation (GGA) in the form proposed by Perdew, Burke, and Ernzerhof (PBE)^33^,^34^. A plane-wave basis set with kinetic energy cutoff of 500 eV is used with energy precision of 1E-5 eV. Periodic boundary conditions are employed to simulate these 2D systems. Vacuum space between a constructed structure and its periodic mirrors is chosen to be 15 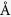, which is sufficient for energy convergence. For the 2D structures, the Brillouin zone (BZ) was sampled by using a 11 × 11 × 1 Gamma-centered Monkhorst-Pack grid^35^, whereas a 1 × 5 × 1 grid was used for the nanoribbon. The atomic coordinates of all atoms in the unit cell and the cell’s length are fully relaxed, using the conjugate gradient (CG) scheme, with the forces on every atom converged to within 0. 01 eV/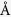. SOC was included by a second variational procedure on a fully self-consistent basis.

## Additional Information

**How to cite this article**: Liu, Q. *et al*. Large gap Quantum Spin Hall Insulators of Hexagonal III-Bi monolayer. *Sci. Rep.* **6**, 34861; doi: 10.1038/srep34861 (2016).

## Figures and Tables

**Table 1:** Lattice constant c_0_ (Å), bulking height h(Å) and bond length l (Å) of XBi_3_.

Structure	C_0_	h	l_BiBi_	l_BiX_
GaBi_3_	8. 824	1. 861	3. 035	2. 777
InBi_3_	9. 121	1. 884	3. 033	2. 923
TlBi_3_	9. 170	1. 976	3. 016	3. 079
